# Family Caregivers’ Experiences and Changes in Caregiving Tasks During the COVID-19 Pandemic

**DOI:** 10.1177/10547738211014211

**Published:** 2021-05-17

**Authors:** Elliane Irani, Atsadaporn Niyomyart, Ronald L. Hickman

**Affiliations:** 1 Frances Payne Bolton School of Nursing, Case Western Reserve University, Cleveland, OH, USA

**Keywords:** family caregiver, COVID-19 pandemic, caregiver burden

## Abstract

The purpose of this descriptive study was to describe family caregivers’ experiences and changes in caregiving tasks and approaches during the COVID-19 pandemic. Using web-based strategies, 69 family caregivers of adults with chronic or disabling conditions were recruited and completed an online survey about positive and negative caregiving experiences, and ways in which caregiving has changed. Data were analyzed using descriptive statistics (structured questions) and conventional content analysis (open-ended responses). Participants reported concerns about their loved one’s physical and mental health, the limited access to other caregiving sources, and the limited opportunities to maintain personal well-being. Caregiving tasks completed more than usual included providing emotional support, shopping for groceries and essentials, and contacting healthcare providers. Participants modified their caregiving approach by assuming added responsibilities, leveraging technology, and managing a new caregiving routine. Findings indicate that family caregivers experienced additional caregiving challenges and changed caregiving tasks considering the limited resources available.

Family caregivers play an essential role in maintaining the health and well-being of individuals with chronic and disabling conditions. They offer emotional and instrumental support and coordinate healthcare services, and nearly half of them often perform skilled tasks such as injections, catheter or colostomy care, wound care, and medical equipment monitoring (Reinhard et al., 2019). As a result of the coronavirus disease 2019 (COVID-19) pandemic, family caregivers are navigating new social restrictions while providing care to their loved ones in the community. They are faced with unanticipated stressors, especially in that they are caring for the most vulnerable group of individuals at increased risk for severe illness from COVID-19 (Tisminetzky et al., 2020). Therefore, it is important to explore how family caregivers are affected by the spread of the severe acute respiratory syndrome coronavirus 2 (SARS-CoV-2) and the associated restrictions that took effect.

During the COVID-19 pandemic, caregiving is associated with additional challenges because family caregivers are facing highly unusual circumstances and a disruption to their caregiving routine. National and local policies implemented to contain the spread of SARS-CoV-2 may have led to a change in caregiving intensity, increased feelings of stress, and limited time for family caregivers to manage their own health. For instance, community-based services such as adult day centers were closed, which limited the amount of support and respite care usually received by family caregivers of community-dwelling persons with dementia (Greenberg et al., 2020). Moreover, the stay-at-home orders resulted in changing demands on younger and/or older members of the family and disrupted social interactions, creating a state of isolation (Usher et al., 2020). During the initial phase of the COVID-19 pandemic, family caregivers were more likely to report psychological distress and fatigue compared to non-caregivers (Park, 2020). Family caregivers’ concerns about the pandemic were associated with a greater perception of role overload, which can negatively influence their psychological well-being (Savla et al., 2021). Lastly, at the healthcare system level, visitation restrictions were instated in many health facilities and there was an abrupt shift to telehealth as a primary care delivery model. These changes resulted in different dynamics between healthcare providers and family caregivers, potentially leaving family caregivers with greater uncertainty around decision making and little support from healthcare professionals they have typically relied upon.

Previous research highlighted several factors that place certain family caregivers at a greater risk for increased burden and negative caregiving experiences (Adelman et al., 2014; Kim et al., 2012). For instance, family caregivers who are socially isolated or have no choice about assuming certain caregiving tasks are more at risk for experiencing difficulties when caring for their loved ones (Reinhard et al., 2019). Therefore, it is anticipated that most family caregivers would report greater caregiving difficulties during the pandemic. Moreover, coresidence with the care recipient plays a role in how family caregivers have adapted their caregiving routines. In fact, those living with the care recipient are more likely to provide a higher intensity of care and often report higher levels of burden (Huang et al., 2020). Therefore, the pandemic-induced restrictions may disproportionately affect family caregivers living with the care recipient.

Family caregivers play critical roles in our society and care for those at increased risk of severe illness from COVID-19. Family caregiving refers to the experiences and activities involved in providing assistance to a family member or friend with health needs (Pearlin et al., 1990). The COVID-19 pandemic has added substantial and unforeseen stressors that may have influenced family caregivers’ experiences and their ability to sufficiently meet the needs of those with chronic conditions. Therefore, the purpose of this study is to describe how family caregivers were affected during the COVID-19 pandemic. Specifically, we aimed to answer the following three research questions:

1. What are the experiences of family caregivers while providing care to their loved one during the pandemic?
2. How have caregiving tasks and approaches changed as a result of the pandemic and its associated restrictions?
3. Are caregivers’ experiences and changes in caregiving tasks associated with coresidence status?

This study will contribute to a further understanding of the needs of family caregivers during the rapidly evolving COVID-19 pandemic. The findings will benefit healthcare providers, researchers, and policy makers through identification of specific areas of intervention to offer family caregivers greater support during and beyond the pandemic.

## Methods

### Design and Sample

This descriptive study involved a convenience sample of 69 family caregivers who participated in an online survey between May and September 2020. The survey included structured and open-ended questions. Data were analyzed using descriptive quantitative and qualitative methods in order to describe the phenomena of interest (i.e., caregivers’ experiences and changes to caregiving tasks).

Family caregivers were invited to participate if they were aged 18 years or older, lived in the United States, provided care for a community-dwelling adult relative or friend with an existing chronic or disabling condition, and were able to speak, read, and understand English. The study qualified for exemption and was approved by the Institutional Review Board at Case Western Reserve University (STUDY20200512).

### Procedures

Two recruitment strategies were used in this study to achieve the final analytic sample. First, participants were recruited through social media (i.e., Facebook caregiving support groups), a professional network of caregiving researchers, and community organizations that serve older adults and their families. The announcements included information about the study, the expected duration of the survey, the inclusion criteria, the contact information for the principal investigator, and a link to the survey for data collection. The second recruitment strategy consisted of snowball sampling. Early participants and caregiving researchers were asked to share the study information with others who may be eligible and interested in taking part in the study.

All data were collected via a Research Electronic Data Capture (REDCap) survey. Participants were directed to the survey questions after reviewing the consent form and verifying their eligibility on the first page of the online survey. As an incentive, study participants who completed the online survey had the option of entering into a drawing for one of four electronic gift cards valued at $25.

### Data Collection

Participants completed questions about their age, gender, race, ethnicity, living arrangements, and other demographic characteristics. They were also asked to report on specific characteristics related to their caregiving role, such as the number of hours per week providing care for their loved one and the number of years as a caregiver. Participants were also asked about their loved ones’ demographic information, level of dependence, and health conditions or diagnoses.

#### Caregiving experiences

Participants were asked to select negative and positive caregiving experiences associated with the pandemic based on a list of 12 statements that were developed by members of the research team and reviewed by an independent expert in caregiving research. There were nine statements representing negative experiences (e.g., worried about getting sick and not being able to care for my loved one) and three statements representing positive experiences (e.g., spending more time with my loved one). Then, participants rated their caregiving stress in comparison with before the pandemic on a 3-point Likert scale (1 = *less stressful than usual* to 3 = *more stressful than usual*). If a participant indicated that caregiving during the pandemic was *more stressful* than usual, they were directed to an open-ended question asking about the caregiving challenges they have experienced. Those who indicated that their caregiving was *less stressful* during the pandemic were invited to share their positive experiences using an open-ended question format. The two open-ended questions were administered to supplement the quantitative survey data and provide a comprehensive representation of the positive and negative caregiving experiences in the context of the pandemic.

#### Caregiving tasks

Participants were asked to indicate how their usual caregiving tasks have changed during the pandemic. They rated how much they were helping their loved one in reference to a list of activities of daily living (ADLs) and instrumental activities of daily living (IADLs) that caregivers often assist with. Answer choices were *more than usual*, *about the same as usual*, and *less than usual*. Lastly, in an open-ended question, participants were asked about ways in which they modified their caregiving approach during the pandemic.

### Data Analysis

The first two research questions were addressed using descriptive statistics and content analysis. The source of data for the first two research questions included the quantitative survey responses about the 12 statements, the ratings about changes in caregiving tasks, and the qualitative responses to the open-ended questions. We first used descriptive statistics to characterize the sample and describe the percentage of participants reporting each of the negative and positive experiences (Research Question 1). The percentage of family caregivers reporting an increase in caregiving tasks was also calculated for each task separately (Research Question 2). We analyzed the qualitative data obtained from the open-ended questions using conventional content analysis because prior knowledge on the phenomenon of interest is not available (Elo & Kyngäs, 2008; Hsieh & Shannon, 2005). Participant responses were reviewed separately for each open-ended question to identify the meaning units that were assigned a code. Then first-level codes were grouped based on commonalities to form the final categories that represent caregiving experiences (Research Question 1), and changes in caregiving approaches (Research Question 2; Graneheim & Lundman, 2004). To ensure rigor, qualitative data were coded by a single researcher and independently reviewed by another researcher to ensure accuracy of coding and categorization (Morse, 2015). An audit trail was also kept to track analytic decisions throughout the analysis process (Koch, 2006). Findings were discussed among members of the research team to reach final consensus. The qualitative findings were used to support or extend the quantitative findings.

Lastly, the third research question was addressed using the Chi-Square Test of Independence to assess whether caregiving experiences and changes in caregiving tasks were associated with coresidence status. Participants were grouped into two categories based on whether or not they were living with the care recipient at the time of answering the survey. Significance level was set at <.05. Quantitative survey data were analyzed using SPSS version 26, and qualitative data from the open-ended questions were analyzed using NVivo 12.

## Results

### Sample Characteristics

The majority of study participants were women (87%) and were on average 54.7 (±13.6) years old. Most of them self-identified as White (71%) and had a college degree (73.9%). Participants were residing in 23 states and about half of them lived in the midwestern United States. Half of our sample provided care to a parent, and two-thirds of the sample lived with the care recipient. On average, participants reported being a family caregiver for 7.7 (±7.7) years. Twenty-nine participants (42%) indicated that they were the only person assisting their loved one with their care needs. The remaining participants (*n* = 40; 58%) reported receiving assistance from other family members or friends. Participants’ characteristics are presented in Table 1.

**Table 1.** Sample Characteristics (*N* = 69).

Variable	*n* (%)
**Gender**	
Female	60 (87.0)
Male	9 (13.0)
**Race**	
Non-White	20 (29.0)
White	49 (71.0)
**Ethnicity**	
Hispanic/Latinx	2 (2.9)
Not Hispanic/Latinx	67 (97.1)
**Marital status**	
Not married	24 (34.8)
Married/in a relationship	45 (65.2)
**Education**	
High school degree	2 (2.9)
Some college (no degree)	11 (15.9)
Associate degree	5 (7.2)
Bachelor’s degree	21 (30.4)
Graduate degree	23 (33.3)
Doctoral degree	7 (10.1)
**Employment status**	
Unemployed	24 (34.8)
Employed	45 (65.2)
**Relationship to care recipient**	
Child	39 (56.5)
Spouse/partner	17 (24.6)
Other family member or friend	13 (18.8)
**Co-residence status**	
Co-residing caregiver	42 (60.9)
Distant caregiver	27 (39.1)
**Caregiving hours per week**	
≤20 hours	37 (53.6)
>20 hours	32 (46.4)
**State of residence** ^a^	
Midwest	37 (53.6)
West	17 (24.6)
South	11 (15.9)
Northeast	3 (4.3)
**Care recipient’s dependence**	
Slightly dependent	17 (24.6)
Dependent	36 (52.2)
Completely dependent	16 (23.2)
**Caregiver income**	
Have more than enough	28 (40.6)
Have enough to make ends meet	34 (49.3)
Do not have enough	7 (10.1)
**Support from other family caregivers**	
Yes	50 (58)
No	29 (42)
**Caregiver social support** ^a^	
Poor	6 (8.7)
Satisfactory	14 (20.3)
Good	28 (40.6)
Very good	13 (18.8)
Excellent	7 (10.1)
**Care recipient’s conditions**	
Cardiovascular disease	45 (65.2)
Arthritis	33 (47.8)
Dementia	23 (33.3)
Diabetes	19 (27.5)
Mental health conditions	14 (20.3)
Obesity	13 (18.8)
Cancer	9 (13.0)
Chronic kidney disease	7 (10.1)
Lung disease	5 (7.2)
Parkinson’s disease	4 (5.8)
**Home- and community-based services received**	
Skilled home health care	13 (18.8)
Adult day care	11 (15.9)
Home-delivered meals	7 (10.1)
Transportation services	10 (14.5)
**Caregiving stress during the pandemic**	
Higher than usual	50 (72.5)
Lower than usual	4 (5.8)
About the same	15 (21.7)

a *n* = 68 for state of residence and caregiver social support.

We present the findings of each research question separately. To address the first two research questions, we will first present the findings based on our analysis of the quantitative survey data, then describe the qualitative findings from the open-ended questions. Lastly, we will address the third research question by describing whether caregiving experiences and tasks are different based on coresidence status.

### Caregiving Experiences

The negative and positive caregiving experiences reported by participants are presented in Table 2 based on the frequency of their reporting. Some participants (36.1%) highlighted positive caregiving experiences, mostly as it relates to being able to spend more time with their loved one. This finding was also highlighted in one of the responses to the open-ended question. One participant indicated that her caregiving experience was less stressful than usual “because [she is] not traveling and [is] spending time away from work.” Four participants reported that their caregiving stress during the pandemic was less than usual. Therefore, there were not enough qualitative responses to conduct content analysis using data from the open-ended question about positive caregiving experiences.

**Table 2.** Experiences of Family Caregivers during the COVID-19 Pandemic.

	Total (*N* = 69)	Co-residing caregivers (*n* = 42)	Non co-residing caregivers (n = 27)	**χ** ^2^
	*n* (%)^a^	*n* (%)^a^	*n* (%)^a^	
**Negative experiences**				
Made me worried about getting sick and not being able to care for my loved one	47 (68.1)	30 (71.4)	17 (63.0)	0.54
Made it difficult for me to shop for groceries and essential items	32 (46.4)	21 (50.0)	11 (40.7)	0.57
Limited my ability to get help from other caregiving sources (such as other family members, community organizations, or formal home care services)	32 (46.4)	20 (47.6)	12 (44.4)	0.07
Limited my ability to be present with my loved one while in the hospital or during healthcare appointments	25 (36.2)	10 (23.8)	15 (55.6)	7.17**
Limited my ability to visit my loved one as frequently as I would like to	19 (27.5)	2 (4.8)	17 (63.0)	27.90***
Made it difficult for me to get the medications and medical supplies that my loved one needs	17 (24.6)	9 (21.4)	8 (29.6)	0.60
Limited my ability to spend time with my loved one	17 (24.6)	1 (2.4)	16 (59.3)	28.64***
Limited my ability to get information from my loved one’s healthcare providers	14 (20.3)	4 (9.5)	10 (37.0)	7.69**
**Positive experiences**				
Allowed me to spend more time with my loved one	18 (26.1)	16 (38.1)	2 (7.4)	8.03**
Connected me with new supports^b^	8 (11.6)	5 (11.9)	3 (11.1)	
Made me less worried about other responsibilities^b^	6 (8.7)	4 (9.5)	2 (7.4)	

a The numbers and percentages correspond to participants affirming the statements about negative and positive experiences.

b The Fisher’s exact test was used when cells had expected counts <5.

** *p* < .01. ***p < .001.

In terms of negative caregiving experiences, the majority (68.1%) of participants voiced a concern about getting sick and not being able to care for their loved one. Nearly half of our participants (46.4%) expressed challenges associated with grocery shopping, as well as their inability to get help from other caregiving sources. These concerns were also highlighted in several responses to the open-ended question. All of the participants who reported a more stressful caregiving experience during the pandemic answered the open-ended question about the challenges they have experienced. Qualitative responses were grouped into two broad categories related to negative caregiving experiences: (1) concerns related to their loved one, and (2) personal concerns.

#### Concerns related to their loved one

In the open-ended responses, participants described challenging experiences directly related to their loved ones’ care, physical health and safety, and mental well-being. One participant said that his caregiving experience is more stressful than usual because he is “unable to actively participate in the care provided by physicians and nurses in office and home settings.” In terms of physical health and safety concerns, participants were worried about transmitting the virus to their loved one or increasing the risk of their loved one being exposed to COVID-19 by interacting with other people. One participant said, “I am an ‘essential worker.’ The concern that I could take the virus home to him is high.” In another response, a participant expressed her fear over her parents’ safety: “[I am] scared when I have to leave them alone and have them interact with neighbors, handle deliveries, etc. and not use protective and cautious measures, and contract COVID.” Lastly, participants were worried that their loved one did not have enough opportunities to remain connected with others and entertained, which had detrimental effects on the loved ones’ mental health and well-being, as illustrated in the following response: “She is used to volunteering three days a week, and without this purpose, she is becoming very depressed.” Other participants explained how their loved one no longer had access to community support groups or adult day cares, and subsequently exhibited more behavioral problems.

#### Personal concerns

Participants reported personal concerns and challenges due to the limited help they received from other caregiving sources, the added responsibilities they needed to manage, and the limited opportunities available to maintain their own well-being. They indicated that most of the home- and community-based services became less available to their loved one or were suspended indefinitely. Other participants also explained that they were no longer receiving support from other family members, as exemplified in the following response: “Before the pandemic, we had other family members who would come in and rotate helping us.” Therefore, participants faced caregiving challenges and needed to manage additional responsibilities. Besides the added caregiving responsibilities, they experienced stress while attending to the needs of other family members and while shopping for groceries. One said, “Access to grocery stores and pharmacies has been possible, but requires more planning and becomes stressful.” Lastly, the majority of participants experienced distress because of having limited opportunities to recharge and maintain their personal well-being. One participant explained, “All physical socialization avenues have been eliminated . . . My caregiving experience is more stressful because I have few outlets. I am home all day every day.” The concern about personal well-being emerged as a culminating effect of the physical distancing restrictions, limited access to help from others, and added responsibilities at all levels.

### Changes in Caregiving Tasks and Approaches

Many participants indicated helping their loved one more than usual with several tasks, including providing emotional support (58%), shopping for groceries or other essentials (52.2%), contacting healthcare providers (49.3%), encouraging activity (47.8%), and preparing meals (46.4%). The list of caregiving tasks that have changed during the pandemic is presented in Table 3.

**Table 3.** Change in Caregiving Tasks during the COVID-19 Pandemic.

Assisting with . . .	Total	Co-residing caregivers	Non-coresiding caregivers	χ^2^
	*n* (%)	*n* (%)	*n* (%)	
Providing emotional support (*n* = 68)				2.47
More than usual	40 (58.0)	21 (51.2)	19 (70.4)	
Same or less than usual	28 (40.6)	20 (48.8)	8 (29.6)	
Shopping (*n* = 65)				5.93*
More than usual	36 (52.2)	18 (43.9)	18 (75.0)	
Same or less than usual	29 (42.0)	23 (56.1)	6 (25.0)	
Contacting providers (*n* = 63)				1.14
More than usual	34 (49.3)	19 (48.7)	15 (62.5)	
Same or less than usual	29 (42.0)	20 (51.3)	9 (37.5)	
Encouraging or helping with being active (*n* = 61)				4.54*
More than usual	33 (47.8)	16 (55.6)	18 (72.0)	
Same or less than usual	28 (40.6)	20 (44.4)	7 (28.0)	
Preparing meals (*n* = 62)				0.12
More than usual	32 (46.4)	20 (50.0)	12 (54.5)	
Same or less than usual	30 (43.5)	20 (50.0)	10 (45.5)	
Getting medications refilled (*n* = 63)				0.22
More than usual	25 (36.2)	15 (37.5)	10 (43.5)	
Same or less than usual	38 (55.1)	25 (62.5)	13 (56.5)	
Reminding about medications (*n* = 50)				0.11
More than usual	21 (30.4)	14 (43.8)	7 (38.9)	
Same or less than usual	29 (42.0)	18 (56.2)	11 (61.1)	
Scheduling healthcare appointments (*n* = 62)				0.01
More than usual	21 (30.4)	13 (33.3)	8 (34.8)	
Same or less than usual	41 (59.4)	26 (66.7)	15 (65.2)	
Handling money (*n* = 56)				1.2
More than usual	19 (27.5)	10 (28.6)	9 (42.9)	
Same or less than usual	37 (53.6)	25 (71.4)	12 (57.1)	
Coordinating services (*n* = 40)				1.76
More than usual	12 (17.4)	5 (21.7)	7 (41.2)	
Same or less than usual	28 (40.6)	18 (78.3)	10 (58.8)	
Eating (*n* = 41)^a^				
More than usual	11 (15.9)	7 (24.1)	4 (33.3)	
Same or less than usual	30 (43.5)	22 (75.9)	8 (66.7)	
Using medical equipment (*n* = 33)^a^				
More than usual	9 (13.0)	4 (21.1)	5 (35.7)	
Same or less than usual	24 (34.8)	15 (78.9)	9 (64.3)	
Dressing (*n* = 39)^a^				
More than usual	8 (11.6)	3 (75.0)	1 (9.1)	
Same or less than usual	31 (44.9)	10 (25.0)	10 (90.9)	
Bathing (*n* = 36)^a^				
More than usual	6 (8.7)	5 (19.2)	1 (10.0)	
Same or less than usual	30 (43.5)	21 (80.8)	9 (90.0)	
Getting in/out of chair (*n* = 38)^a^				
More than usual	6 (8.7)	4 (18.2)	2 (12.5)	
Same or less than usual	32 (46.4)	18 (81.8)	14 (87.5)	
Walking across a room (*n* = 24)^a^				
More than usual	4 (5.8)	3 (23.1)	1 (9.1)	
Same or less than usual	20 (29.0)	10 (76.9)	10 (90.9)	
Doing wound or ostomy care (*n* = 18)^a^				
More than usual	3 (4.3)	3 (30.0)	0	
Same or less than usual	15 (21.7)	7 (70.0)	8 (100)	

a The Fisher’s exact test was used when cells had expected counts <5.

* *p* < .05.

The responses to the open-ended question were grouped into three categories to illustrate how participants modified their caregiving approach during the pandemic: (1) assuming added responsibilities, (2) leveraging technology, and (3) managing the new caregiving routine.

#### Assuming added responsibilities

The majority of participants described how their caregiving responsibilities have increased during the pandemic, specifically as it relates to minimizing the risk of COVID-19 infection, providing their loved one with continuous emotional support, and taking on additional skilled tasks. To minimize the risk of their loved one from getting COVID-19, participants became responsible for shopping and preparing meals. They needed to remain vigilant at all times and identify safe practices and the best times to go to the stores. One participant explained, “I disinfect EVERYTHING that I bring in the house after shopping. This process is time-consuming but necessary, given all of the COVID-19 unknowns. I also disinfect the mail . . . I never had to do these things prior to the pandemic.” Participants also indicated spending time to educate their loved one about the virus and the importance of wearing a mask and remaining physically distant from others. Some participants described how they limited the in-person contact of their loved one with others to avoid potential exposure to the virus.

Besides minimizing the risk of COVID-19 infection, participants provided regular and intensive emotional support to their loved one to offset the psychological impact of the distancing restrictions. Some participants decided to modify their loved ones’ living arrangements, as exemplified in one’s remark: “Instead of periodic emotional support and supervision, I am providing constant emotional and mental health care and co-living because he is scared of the COVID situation and refuses to live alone.”

Lastly, participants took on significantly more responsibility for skilled tasks and routine supervision because of the limited access to other support systems and resources. One participant explained, “My husband has a PICC line to receive IV antibiotics. During this pandemic, I now draw blood to deliver to the hospital.” In other cases, participants described how they needed to encourage their loved one more than usual to be active and independent in their activities of daily living. Participants who stopped receiving support from other caregiving sources often became the sole caregiver and had to monitor their loved one more frequently to avoid any unsafe behavior or adverse events.

#### Leveraging technology

Participants described how they used technology to keep their loved one connected with other friends and family members. Some needed to provide additional guidance to their loved one given the technological challenges that older people may face. One participant said, “The virtual world is great, if your elderly parents can actually access it . . . He can’t even attend AA (Alcoholics Anonymous) meetings because he can’t use Zoom effectively on his own.” Participants also explained how technology facilitated their communication with their loved ones’ healthcare providers, specifically during virtual health visits. They appreciated the benefit of virtually meeting providers while decreasing the risk of exposure to the virus.

#### Managing the new caregiving routine

Participants reported the need to adopt a new caregiving routine during the pandemic, as represented in the following response: “I have to adapt caregiving around the fact that I am working from home. No boundaries between work and home.” They used strategies to navigate and manage the added caregiving responsibilities while maintaining their overall level of functioning. These strategies included keeping a schedule for their loved one, prioritizing their tasks, and being less worried about other responsibilities. For example, one participant explained, “I had to readjust my work schedule, make more time for breaks to care for her needs, especially when she needed social and emotional support.” Another participant described her decision about eliminating some tasks: “I allow the voicemail to catch calls and return them at a time which is good for me . . . I am not busting myself up if I can’t get something done.” On the other hand, some participants reported experiencing a higher level of stress because they were not able to manage the added responsibilities and did not have any personal time. In some cases, the increased stress level led participants to become easily irritated and less compassionate toward their loved ones.

### Coresidence Status and Caregiving During the Pandemic

There was a statistically significant association between participants’ coresidence status and reporting caregiving challenges related to spending time with their loved ones (χ^2^ (1) = 28.64, *p* < .001), visiting them in their home (χ^2^ (1) = 27.90, *p* < .001), getting information from healthcare providers (χ^2^ (1) = 7.69, *p* = .006), and being physically present during healthcare encounters (χ^2^ (1) = 7.17, *p* = .007). Compared to participants residing with the care recipients, non-coresiding family caregivers reported a significantly higher percentage of challenges related to spending time with their loved ones (94.1% vs. 5.9%), visiting them (89.5% vs. 10.1%), getting information from their healthcare providers (71.4% vs. 28.6%), and being physically present during their healthcare encounters (60% vs. 40%). There was a statistically significant association between coresidence status and reporting the benefit of spending more time with their loved ones (χ^2^(1) = 8.03, *p* = .005). Among participants reporting spending more time with their loved ones, more individuals significantly belonged to the coresiding group (88.9%) as compared to the non-coresiding group (11.1%).

Lastly, there was a statistically significant association between coresidence status and helping their loved ones more than usual by shopping for groceries or other essentials (χ^2^ (1) = 5.93, *p* = .015) and encouraging them to exercise or be active (χ^2^(1) = 4.54, *p* = .033). Among participants who do not live with the care recipient, a greater percentage reported helping their loved ones more than usual with grocery shopping (75%) and encouraging them to exercise and remain active (72%), as compared to helping as usual or less than usual with grocery shopping (25%) and remaining active (28%).

## Discussion

The purpose of our study was to describe the experiences of family caregivers during the COVID-19 pandemic and ascertain how the pandemic-related restrictions affected their day-to-day lives, including their caregiving tasks and approaches. Family caregivers are facing numerous challenges due to the limited access to other caregiving sources and their concerns about their loved ones’ physical and mental health. Family caregivers are assuming added responsibilities and adapting to their new caregiving routine. Our findings highlight areas for intervention to support family caregivers during and beyond the pandemic.

Our participants described the challenges associated with receiving less assistance from other caregiving sources, which led them to assume new caregiving responsibilities. Our results are consistent with those of two recent studies that focused on family caregivers of persons with dementia. In the first study, Savla et al. (2021) found that a third of family caregivers were not receiving support from other family members, and some were concerned about being the sole caregivers. Moreover, many of their participants had some level of burnout because home health services were reduced or stopped. This finding was echoed in the second study, in which Cohen et al. (2020) found that family caregivers of persons with advanced dementia were most concerned about the paid caregivers no longer assisting with their loved ones’ care. While these two studies focused on a different population of family caregivers, the concerns about limited caregiving support reflect a universal experience of family caregivers during the COVID-19 pandemic and require timely intervention.

Our participants also reported several concerns related to maintaining their loved ones’ physical health, safety, and mental well-being. The Centers for Disease Control and Prevention (2020) has recommended several preventive measures to contain the spread of SARS-CoV-2 and avoid health complications in people who are most at risk. Older people living in community settings are recommended to stay home as much as possible, and when outside, to practice social distancing in combination with other preventive actions such as wearing masks. Consistent with our findings, family caregivers of persons with dementia are concerned about their loved ones following the recommendations to remain safe and healthy, and are frustrated about the limited opportunities for these care recipients to remain socially connected and engaged in enjoyable activities (Savla et al., 2021). Others reported a concern about increasing the risk of COVID-19 transmission while assisting their loved ones with care needs (Cohen et al., 2020). It is important for family caregivers to remain informed about strategies to follow the guidelines and reduce the distress associated with the pandemic restrictions. For instance, Greenberg et al. (2020) recommended that caregivers of persons with dementia model good hygiene practices and role-play a favorite character wearing a mask. Other suggestions include engaging in personalized activities based on interest and cognitive ability or using technology to stay connected with others (Lightfoot & Moone, 2020). However, the use of technology remains difficult for many older adults, as illustrated in our findings, and many families may not have access to the internet. More needs to be done before recommending technologically-mediated interactions across the board to mitigate the social impact of the pandemic.

Even under usual circumstances, family caregiving has been linked to poor health and well-being (Schulz et al., 2020). These adverse health outcomes may have been exacerbated during the pandemic and will become of greater concern as the pandemic period extends. Several participants described taking on additional responsibilities and adjusting to their new caregiving routine as a result of the evolving pandemic-related restrictions put in place to keep communities safe. It may be difficult for caregivers to perceive caregiving gains or positive experiences when they did not have a choice about increasing their caregiving intensity during the pandemic. Moreover, these caregivers have fewer opportunities to recharge and maintain their well-being, as illustrated in some of the open-ended responses of the participants in our study. Therefore, family caregivers should use personal strategies to overcome the disruption to their routine and adopt new caregiving approaches. For example, the health benefits of gratitude are well-known (Jans-Beken et al., 2020) and have been recently established in the context of COVID-19 (Jiang, 2020). Individuals feeling more gratitude than average had a lower perception of stress related to COVID-19, a higher level of positive affect, and a lower level of negative affect (Jiang, 2020). Additional research is needed to examine if a similar association exists for family caregivers. Subsequently, gratitude can be recommended as a simple stress-reduction strategy to offset the effects of caregiving stress during the pandemic and improve emotional well-being among family caregivers.

Our findings have several implications for research, practice, and policy to support family caregivers during and beyond the pandemic. Future research studies are needed to identify the long-term effects of the pandemic on the health of family caregivers and their caregiving capacity. While some of our participants shared examples of successfully prioritizing their tasks and managing multiple responsibilities, others struggled to manage the added stressors. Researchers need to target family caregivers who report sustained levels of high stress and are unable to adapt to their new routine. Existing self-regulation and stress management interventions can be tailored to the context of the pandemic to benefit all family caregivers, and particularly those experiencing higher levels of stress.

Our findings have practice implications for nurses as well, specifically those in community-based settings, to direct family caregivers to the needed resources. Since the beginning of the pandemic, family caregivers have had a major disruption to their routine and are now navigating a healthcare system that may not be responsive enough to their non-acute care needs. Nurses can assist family caregivers in identifying the source of their stress and adopting creative strategies to address their challenges. For example, nurses can recommend those who are worried about shopping for food and essential items to take advantage of local delivery services or delegate some tasks to other family members. Moreover, nurses can play a role in better educating family caregivers about practicing physical distancing while remaining socially engaged with others to avoid contracting COVID-19 without compromising their psychological well-being. Nurses also take into consideration other contextual factors that can influence the caregiving experience. We found that family caregivers have different concerns based on their coresidence status. For instance, participants who did not live with the care recipients reported their inability to visit their loved ones and spend time with them as a major challenge. Nurses can build on our findings and explore other influencing social factors as they provide recommendations to family caregivers.

Lastly, our findings underscore the need for policies aimed at supporting family caregivers in their role during and beyond the pandemic. Policies that benefit caregivers and families in need have the potential to improve the general well-being of the nation (Stokes & Patterson, 2020). For example, working family caregivers may be unable to afford leaving their jobs and would benefit from a paid family leave to decrease the risk of transmitting COVID-19 to their loved ones while maintaining their employment status. Since the beginning of the pandemic, some states have implemented flexibilities available through emergency waiver programs that can allow family caregivers of Medicaid beneficiaries to receive reimbursement for providing specific home- and community-based services (Kaye & Teshale, 2020). These flexibilities have been implemented in response to the pandemic, but can serve as a foundation for future and possibly permanent state policies. It is important for family caregivers to receive timely and accurate updates about local, state, and national policies pertaining to family caregiving. Family caregivers are also encouraged to take action and be involved in advocacy efforts through community-based organizations to raise awareness about their specific needs during the pandemic and get access to additional services.

The findings of this study need to be considered in light of some limitations. Our findings may not represent the experiences of the broader population of family caregivers. Our participants were all English-speaking, living in the United States, mostly highly educated, and with access to the internet. Other family caregivers with more diverse backgrounds may report additional challenges or different perspectives on how their caregiving tasks have changed. Moreover, our data collection methods may have influenced participants’ responses on the open-ended questions, which were the primary source of qualitative data in this study. For instance, data about caregiving challenges and positive experiences were only gathered from participants who reported a higher or a lower level of caregiving stress during the pandemic, respectively. Regardless of their stress level, family caregivers may have had positive and challenging experiences that were overlooked in this study. Additional qualitative research using semi-structured interviews is needed to gain an in-depth understanding of the challenges and positive caregiving experiences. Lastly, our results represent the experiences of family caregivers caring for individuals with diverse chronic and/or disabling conditions. Existing research has shown that caregiving intensity and burden depend on the health conditions of care recipients (Zauszniewski et al., 2020). Future research is needed to examine whether caregiving experiences and changes to caregiving tasks are different based on care recipients’ health conditions. Despite these limitations, our study is among the first to describe the overall experiences of family caregivers of adults with chronic illness during the COVID-19 pandemic.

## Conclusion

In this paper, we described the challenges and positive experiences of family caregivers and summarized how caregiving tasks and approaches have changed as a result of the rapidly evolving COVID-19 pandemic. Family caregivers experienced increased levels of stress and additional caregiving challenges during the pandemic. They were concerned about their loved ones’ physical and mental health, and had limited opportunities to maintain their own well-being. They needed to assume several added responsibilities within a short period of time, in light of the limited access to other sources of support. The challenges experienced by family caregivers during the pandemic will have long-lasting effects on their health and functioning in the absence of early interventions. As COVID-19 vaccines become more widely available and people around the world progress to recover from the effects of the pandemic, special consideration needs to be given to family caregivers who have faced highly unusual caregiving circumstances.

## References

[bibr1-10547738211014211] Adelman R. D. Tmanova L. L. Delgado D. Dion S. Lachs M. S. (2014). Caregiver burden: A clinical review. JAMA, 311(10), 1052–1060. 10.1001/jama.2014.304 24618967

[bibr2-10547738211014211] Centers for Disease Control and Prevention. (2020, December 13). Older adults: Coronavirus disease 2019 (COVID-19). https://www.cdc.gov/coronavirus/2019-ncov/need-extra-precautions/older-adults.html

[bibr3-10547738211014211] Cohen G. Russo M. J. Campos J. A. Allegri R. F. (2020). Living with dementia: Increased level of caregiver stress in times of COVID-19. International Psychogeriatrics, 32(11), 1377–1381. 10.1017/S1041610220001593 32729446 PMC7453351

[bibr4-10547738211014211] Elo S. Kyngäs H. (2008). The qualitative content analysis process. Journal of Advanced Nursing, 62(1), 107–115. 18352969 10.1111/j.1365-2648.2007.04569.x

[bibr5-10547738211014211] Graneheim U. H. Lundman B. (2004). Qualitative content analysis in nursing research: Concepts, procedures and measures to achieve trustworthiness. Nurse Education Today, 24(2), 105–112. 14769454 10.1016/j.nedt.2003.10.001

[bibr6-10547738211014211] Greenberg N. E. Wallick A. Brown L. M. (2020). Impact of COVID-19 pandemic restrictions on community-dwelling caregivers and persons with dementia. Psychological Trauma: Theory, Research, Practice, and Policy, 12(S1), S220–s221. 10.1037/tra0000793 32584105

[bibr7-10547738211014211] Hsieh H. F. Shannon S. E. (2005). Three approaches to qualitative content analysis. Qualitative Health Research, 15(9), 1277–1288. 10.1177/1049732305276687 16204405

[bibr8-10547738211014211] Huang J. Chau P. H. Choi E. P. H. Wu B. Lou V. W. Q. (2020). The patterns of caregiving activities for family caregivers of older adults in Hong Kong: An exploratory latent class analysis. The Journals of Gerontology. Series B, Psychological Sciences and Social Sciences. Advance online publication. 10.1093/geronb/gbaa203 33211887

[bibr9-10547738211014211] Jans-Beken L. Jacobs N. Janssens M. Peeters S. Reijnders J. Lechner L. Lataster J. (2020). Gratitude and health: An updated review. The Journal of Positive Psychology, 15(6), 743–782.

[bibr10-10547738211014211] Jiang D. (2020). Feeling gratitude is associated with better well-being across the life span: A daily diary study during the COVID-19 outbreak. The Journals of Gerontology: Series B, Psychological Sciences and Social Sciences. Advance online publication. 10.1093/geronb/gbaa220 PMC7798492 33284976

[bibr11-10547738211014211] Kaye N. Teshale S. (2020, October 29). Medicaid supports for family caregivers. The National Academy for State Health Policy. https://www.nashp.org/medicaid-supports-for-family-caregivers/#toggle-id-7-closed

[bibr12-10547738211014211] Kim H. Chang M. Rose K. Kim S. (2012). Predictors of caregiver burden in caregivers of individuals with dementia. Journal of Advanced Nursing, 68(4), 846–855. 10.1111/j.1365-2648.2011.05787.x 21793872

[bibr13-10547738211014211] Koch T. (2006). Establishing rigour in qualitative research: the decision trail. Journal of Advanced Nursing, 53(1), 91–100. 16422698 10.1111/j.1365-2648.2006.03681.x

[bibr14-10547738211014211] Lightfoot E. Moone R. P. (2020). Caregiving in times of uncertainty: Helping adult children of aging parents find support during the COVID-19 outbreak. Journal of Gerontological Social Work, 63(6–7), 542–552. 10.1080/01634372.2020.1769793 32449648

[bibr15-10547738211014211] Morse J. M. (2015). Critical analysis of strategies for determining rigor in qualitative inquiry. Qualitative Health Research, 25(9), 1212–1222. 10.1177/1049732315588501 26184336

[bibr16-10547738211014211] Park S. S. (2020). Caregivers’ mental health and somatic symptoms during Covid-19. The Journals of Gerontology: Series B, Psychological Sciences and Social Sciences, 76(4), e235–e240. 10.1093/geronb/gbaa121 PMC7454918 32738144

[bibr17-10547738211014211] Pearlin L. I. Mullan J. T. Semple S. J. Skaff M. M. (1990). Caregiving and the stress process: An overview of concepts and their measures. The Gerontologist, 30(5), 583–594. 2276631 10.1093/geront/30.5.583

[bibr18-10547738211014211] Reinhard S. C. Young H. M. Levine C. Kelly K. Choula R. B. Accius J. C. (2019, April). Home alone revisited: Family caregivers providing complex care. AARP Public Policy Institute. 10.26419/ppi.00086.001

[bibr19-10547738211014211] Savla J. Roberto K. A. Blieszner R. McCann B. R. Hoyt E. Knight A. L. (2021). Dementia caregiving during the “stay-at-home” phase of COVID-19 pandemic. The Journals of Gerontology: Series B, Psychological Sciences and Social Sciences, 76(4), e241–e245. 10.1093/geronb/gbaa129 PMC7546083 32827214

[bibr20-10547738211014211] Schulz R. Beach S. R. Czaja S. J. Martire L. M. Monin J. K. (2020). Family caregiving for older adults. Annual Review of Psychology, 71(1), 635–659. 10.1146/annurev-psych-010419-050754 PMC7291827 31905111

[bibr21-10547738211014211] Stokes J. E. Patterson S. E. (2020). Intergenerational relationships, family caregiving policy, and COVID-19 in the United States. Journal of Aging & Social Policy, 32(4–5), 416–424. 10.1080/08959420.2020.1770031 32489144 PMC7754249

[bibr22-10547738211014211] Tisminetzky M. Delude C. Hebert T. Carr C. Goldberg R. J. Gurwitz J. H. (2020). Age, multiple chronic conditions, and COVID-19: A literature review. The Journals of Gerontology Series A, Biological sciences and Medical Sciences. Advance online publication. 10.1093/gerona/glaa320 PMC7799222 33367606

[bibr23-10547738211014211] Usher K. Bhullar N. Jackson D. (2020). Life in the pandemic: Social isolation and mental health. Journal of Clinical Nursing, 29(15–16), 2756–2757. 10.1111/jocn.15290 32250493

[bibr24-10547738211014211] Zauszniewski J. A. Lekhak N. Herbell K. Badr H. (2020). Caregivers of persons with diverse health conditions: Demographics and burden of care. Western Journal of Nursing Research. Advance online publication. 10.1177/0193945920948469 32755293

